# Synthesis, Characterization and Role of Anions (Nitrate, Sulphate, Oxalate and Acetate) \in the Biological Activity of Hydrazine Derived Compounds and Their Metal Chelates

**DOI:** 10.1155/MBD.1997.327

**Published:** 1997

**Authors:** Zahid H. Chohan, S. K. A. Sherazi

**Affiliations:** Department of Chemistry Islamia University Bahawalpur Pakistan

## Abstract

Hydrazine derived compounds and its Co(II), Cu(II) and Ni(II) chelates having the same metal ion but different anions (e.g., nitrate, sulphate, oxalate and acetate) have been synthesised and characterised by their physical, spectral and analytical data. In order to evaluate the participating role of anions in the antibacterial activity, these synthesised ligands and its metal chelates have been screened against bacterial species such as *Staphylococcus aureous, pseudomonas aeruginosa, Klebsiella pneumonae* and *Proteus vulgarus*, and the results are reported.


### SYNTHESIS, CHARACTERIZATION AND ROLE OF ANIONS (NITRATE, SULPHATE, OXALATE AND ACETATE) \IN THE BIOLOGICAL ACTIVITY OF HYDRAZINE DERIVED COMPOUNDS AND THEIR METAL CHELATES

Zahid H. Chohan* and S. K. A. Sherazi

Department of Chemistry, Islamia University, Bahawalpur, Pakistan

### ABSTRACT
Hydrazine derived compounds and its Co(II), Cu(II) and Ni(II) chelates having the same metal ion but different anions (e.g., nitrate, sulphate, oxalate and acetate) have been synthesised and characterised by their physical, spectral and analytical data. In order to evaluate the participating role of anions in the antibacterial activity, these synthesised ligands and its metal chelates have been screened against bacterial species such as *Staphylococcus aureous, Pseudomonas aeruginosa, Klebsiella pneumonae* and *Proteus vulgarus*, and the results are reported.

### INTRODUCTION
Recently, there has been a considerable interest in the chemistry of hydrazine and hydrazone compounds because of their potential pharmacological applications[1-4]. Several reports[5-8] on the metallic chemistry of acyl- and arylhydrazines have already been published. As a further contribution, we have previously reported[9-11] pyrrolyl-, thienyl-, furanyl and benzimidazole-derived hydrazines and their 3d metal chelates which have shown the biological properties. This growing interest between the relationship[12-16] of metals and biological processes has drawn attention[17-20] of many researchers that metals are assential for life as well as for various biochemical reactions.

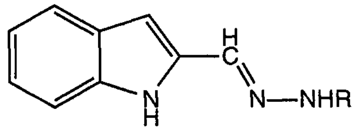

Fig. 1: Structures of the Ligands ($L_1$: R = Ph; $L_2$: R = H)

A number of such biochemical reactions are catalysed[17,18] by enzymes containing metals such as zinc, cobalt, iron etc. Also many metal chelates are found[15,16] to be more carcinostatic than the unchelated compounds/drugs and similarly, many antibacterial drugs when are chelated, their biological activity is effectively altered[7-10]. In understanding the apparent role of metals and their mode of action in biological processes, we have commenced a research program which has already revealed[21-26] the significant role of metals in antibacterial activity whereas, the role of anions which stay as a counterpart of the metals in such chelated compounds has been ignored or not studied before. In this effort to highlight the participating role of anions in biological processes, we wish to report the synthesis, structural and biological studies of Co(II), Cu(II) and Ni(II) metal chelates of hydrazine derived compounds having the same metal atom but different anions (e.g., nitrate, sulphate, oxalate or acetate).

### EXPERIMENTAL
### Material and methods
All chemicals and solvents used were of Analar grade. All the metals were used as their metal(II) salts. Infrared spectra were recorded on Philips Analytical PU 9800 FTIR and Nicolet FTIR instruments. UV-visible spectra were obtained on a Hitachi U-2000 double-beam Spectrophotometers. Conductance of the metal complexes was determined in DMF on a YSI-32 model conductometer. Melting points were recorded on Gallenkamp apparatus and are uncorrected. $^1$H-NMR and $^{13}$C-NMR spectra of the ligands were obtained in DMSO-$d_6$ on a Brucker 250 MHz instrument. The spectral assignments were made by comparing the values with the reported[27] identical compounds. Magnetic measurements were done on solid complexes using the Gouy method. The antibacterial studies were carried out with the help of the Department of Pathology, Quaid-e-Azam Medical College, Bahawalpur (Pakistan).

---

*Present address for correspondence : Department of Chemistry, Meston Walk, University of Aberdeen, Old Aberdeen AB9 2UE, Scotland (U.K)

**Preparation of hydrazine derived compounds**
The title compounds (Fig 1) were prepared by the same method reported[12] earlier by us. Table 1 shows their physical, spectral and analytical data.

**Table 1** Physical, Spectral and Analytical Data of the Ligands

| Ligand/ Mol.Form | M.P (°C) | IR($cm^{-1}$) | $^1$H-NMR(ppm) | $^{13}$C-NMR(ppm) |
|---|---|---|---|---|
| $L_1$ $C_{15}H_{13}N_3$ C:76.61 (calc.77.03) H: 5.52 (5.28) N: 17.86 (17.57) | 175 | 3215,3190,3100, 2920,2516,2020, 1625,1545,1460, 1345,1211,1135, 950 | 4.64(s,1H,arom.), 6.1(s,1H,azomethine), 8.37(s,2H,NH),7.45-7.48(m,3H,*m,p*-Ph), 8.85-8.87(m,2H,*o*-Ph) | 108.51($C_3$),112.29($C_8$), 115.7(*o*-Ph),120.61 (*p*-Ph),121.6($C_5$),123.48($C_6$), 124.75($C_7$),128.0($C_4$), 129.57(*m*-Ph),152.42 (*ipso*),156.22($C_9$),158.1($C_2$), 165.7(azomethine) |
| $L_2$ $C_9H_8N_3$ C:68.37 (68.72) H: 5.06 (5.11) N: 28.56 (26.38) | 158 | 3215,3190,3100, 2925,2015,1625, 1545,1135,955 | 4.63(s,1H,aromatic), 6.7(s,1H,azomethine), 8.34(s,1H,NH),7.18-7.29(m,2H,aromatic), 7.45(m,2H,aromatic), 8.85(s,2H,$NH_2$) | 108.48($C_3$),112.16($C_8$), 121.67($C_5$),123.47($C_5$), 124.77($C_7$),128.1($C_4$), 156.22($C_9$), 158.16 ($C_2$),165.73(azomethine) |

**Preparation of metal complexes**
To a hot ethanolic solution (20 mL) of the ligand (0. 02 mol) was added an aqueous solution (10 mL) of the respective metal(II) (0. 01 mol) salt. The mixture was refluxed for 1h. The resulting mixture was cooled, filtered and reduced nearly half its volume. This concentrated solution was left overnight at room temperature which resulted in the formation of a solid product. The product thus obtained was filtered, washed with ethanol (2x10 mL) then with ether (10 mL) and dried. Crystallisation in hot aqueous ethanol gave the desired complexes 1-24 (Table 2).

**Antibacterial studies**
The synthesised metal chelates and the free ligands were screened for their antibacterial activity against bacterial species, *Staphylococcus aureus(a), Pseudomonas aeruginosa(b), Klebsiella pneumonae(c)* and *Proteus vulgarus(d).* The paper disc diffusion method was used for the determination of antibacterial activity.

**Preparation of discs**
A ligand/complex (30μg) in DMF (0. 01 ml) was applied on a paper disc prepared from blotting paper (3 mm diameter) with the help of a micropipette. The discs were left in an incubator for 48 h at 37°C and then applied on bacteria grown agar plates

**Preparation of agar plates**
Minimal agar was used for the growth of specific bacterial species. For the preparation of agar plates the specific agar was suspended in freshly distilled water (1 L). It was allowed to soak for 15 minutes and then boiled on a water bath until the agar was completely dissolved. The mixture was autoclaved for 15 minutes at 120°C and then poured into previously washed and sterilised petri dishes and stored at 40°C for inoculation.

**Procedure of inoculation**
Inoculation was done with the help of a platinum wire loop which was made red hot on a flame, cooled and then used for the application of bacterial strains.

**Application of discs**
A sterilised forcep was used for the application of paper disc on the already inoculated agar plates. When the discs were applied, they were incubated at 37° C for 24 h. The zone of inhibition was then measured (in diameter) around the disc.

### RESULTS AND DISCUSSION

**Physical properties**
All the metal chelates were found to be coloured, moisture and air stable solids. They are soluble in DMF, DMSO and water and partially soluble in chloroform, acetone, ethanol and benzene. Their melting behaviour, solubility and crystalline nature suggested that they are all non-polymeric.

**Table 2** Physical Data of Metal Chelates

| No | Mol. Formula | | Yield(%) | M.P(°C) (Decomp) | B.M($\mu_{eff}$) |
|---|---|---|---|---|---|
| 1 | $[Co(L_1)_2(NO_3)_2]$ | $C_{30}H_{22}CoN_8O_6$ | 53 | 214-216 | 4.48 |
| 2 | $[Co(L_1)_2(SO_4)]$ | $C_{30}H_{22}CoN_6O_4S$ | 57 | 209-211 | 4.57 |
| 3 | $[Co(L_1)_2(C_2O_4)]$ | $C_{32}H_{22}CoN_6O_4$ | 55 | 198-200 | 4.52 |
| 4 | $[Co(L_1)_2(CH_3CO_2)_2]$ | $C_{34}H_{28}CoN_6O_4$ | 58 | 218-220 | 4.71 |
| 5 | $[Co(L_2)_2(NO_3)_2]$ | $C_{18}H_{14}CoN_8O_6$ | 55 | 197-199 | 4.85 |
| 6 | $[Co(L_2)_2(SO_4)]$ | $C_{18}H_{14}CoN_6O_4S$ | 57 | 202-204 | 4.65 |
| 7 | $[Co(L_2)_2(C_2O_4)]$ | $C_{20}H_{14}CoN_6O_4$ | 53 | 187-189 | 4,55 |
| 8 | $[Co(L_2)_2(CH_3CO_2)_2]$ | $C_{22}H_{20}CoN_6O_4$ | 57 | 200-202 | 4.81 |
| 9 | $[Cu(L_1)_2(NO_3)_2]$ | $C_{30}H_{22}CuN_8O_6$ | 56 | 218-220 | 1.82 |
| 10 | $[Cu(L_1)_2(SO_4)]$ | $C_{30}H_{22}CuN_6O_4S$ | 55 | 223-225 | 1.91 |
| 11 | $[Cu(L_1)_2(C_2O_4)]$ | $C_{32}H_{22}CuN_6O_4$ | 52 | 227-229 | 1.97 |
| 12 | $[Cu(L_1)_2(CH_3CO_2)_2]$ | $C_{34}H_{28}CuN_6O_4$ | 56 | 216-218 | 2.09 |
| 13 | $[Cu(L_2)_2(NO_3)_2]$ | $C_{18}H_{14}CuN_8O_6$ | 55 | 210-212 | 2.28 |
| 14 | $[Cu(L_2)_2(SO_4)]$ | $C_{18}H_{14}CuN_6O_4S$ | 57 | 202-204 | 1.95 |
| 15 | $[Cu(L_2)_2C_2O_4)]$ | $C_{20}H_{14}CuN_6O_4$ | 53 | 211-213 | 1.88 |
| 16 | $[Cu(L_2)_2(CH_3CO_2)_2]$ | $C_{22}H_{20}CuN_6O_4$ | 58 | 218-220 | 2.18 |
| 17 | $[Ni(L_1)_2(NO_3)_2]$ | $C_{30}H_{22}NiN_8O_6$ | 55 | 228-230 | 3.14 |
| 18 | $[Ni(L_1)_2(SO_4)]$ | $C_{30}H_{22}NiN_6O_4S$ | 57 | 234-236 | 3.58 |
| 19 | $[Ni(L_1)_2(C_2O_4)]$ | $C_{32}H_{22}NiN_6O_4$ | 55 | 222-224 | 3.61 |
| 20 | $[Ni(L_1)_2(CH_3CO_2)_2]$ | $C_{34}H_{28}NiN_6O_4$ | 53 | 215-217 | 3.75 |
| 21 | $[Ni(L_2)_2(NO_3)_2]$ | $C_{18}H_{14}NiN_8O_6$ | 55 | 210-212 | 3.38 |
| 22 | $[Ni(L_2)_2(SO_4)]$ | $C_{18}H_{14}NiN_6O_4S$ | 56 | 198-200 | 3.71 |
| 23 | $[Ni(L_2)_2(C_2O_4)]$ | $C_{20}H_{14}NiN_6O_4$ | 57 | 202-204 | 3.58 |
| 24 | $[Ni(L_2)_2(CH_3CO_2)_2]$ | $C_{22}H_{20}NiN_6O_4$ | 55 | 218-220 | 3.63 |

The low molar conductance values (11-15 $ohm^{-1}$ $cm^2$ $mol^{-1}$) of the metal chelates indicated[28,29] that they are all non-electrolyte in nature, probably due to the charge neutralisation of the the metal ion. The room temperature magnetic susceptibility measurements (Table 2) on the solid complexes indicated three unpaired electrons per Co(II) ion (4.48-4.86 B.M), one unpaired electron per Cu(II) ion (1.82-2.28 B.M) and two unpaired electron per Ni(II) ion (3.14-3.75 B.M) which strongly suggested[30,31] octahedral geometry for Co(II) and Ni(II) chelates and distorted octahedral environment for Cu(II) chelates.

**Infrared spectra**

The comparative studies of the important infrared bands of the ligands and its complexes indicated that the ligands are co-ordinated to the metal atom possibly in three ways., a) The ($NH_2$) and (NH) bands appeared at 3215 and 3190 and 3100 $cm^{-1}$ in the case of the free ligand. A lowering in these bands (~ 10-25 $cm^{-1}$) is observed in the case of their metal chelates, indicating[32] co-ordination through these groups.

b) The azomethine (C=N) stretching vibrations are found at 1625 $cm^{-1}$ ; this is also lowered (~ 10-15 $cm^{-1}$) in the case of metal chelates, suggesting co-ordination through nitrogen of the azomethine linkage.

Co-ordination of the metals through the nitrogen donor sites of the ligand was further confirmed by the appearance of the new bands in the region 480-520 $cm^{-1}$, which could be assigned[33] to the respective M-N frequency.

**UV-visible spectra**

The UV-visible spectra of the cobalt chelates show three bands at 9318-9550, 15645-16739 and 21308-22258 $cm^{-1}$ assigned to the transitions $^4T_{1g}$ (F) → $^4T_{2g}$(F)($V_1$), $^4T_{1g}$(F) → $^4A_{2g}$(F)($V_2$) and $^4T_{1g}$(F) → $^4T_{1g}$(P)($V_3$) respectively, and are suggestive[34,35] for octahedral geometry around the cobalt ion. The copper(II) chelates show bands in the region 14510-15162 $cm^{-1}$ , probably due to the $^2E_g$ → $^2T_{2g}$ transitions and the other two bands at 22485-22618 and 30922-31285 $cm^{-1}$ may be are due to intra-ligand charge transfer and d-d transitions for their distorted octahedral environment[36,37].

**Table 3** Spectral and Analytical Data of Metal Chelates

| No | IR ($cm^{-1}$) | $\lambda_{max}$ ($cm^{-1}$) | Calc(Found) % C H N |
|---|---|---|---|
| 1 | 3205($NH_2$),3165,3075(NH), 1610(C=N),480(M-N) | 9318,15645,21308 | 55.49 3.38 17.25 (55.63) (3.11) (17.47) |
| 2 | 3190($NH_2$),3168,3090(NH), 1615(C=N), 488(M-N) | 9455,15811,21675 | 57.99 3.54 13.52 (58.28) (3.77) (13.41) |
| 3 | 3195($NH_2$),3180,3080(NH), 1612(C=N), 495(M-N) | 9550,15650,22258 | 62.66 3.58 13.69 (62.83) (3.39) (13.87) |
| 4 | 3202($NH_2$,3175,3085(NH), 1612(C=N), 520(M-N) | 9480,15896,21882 | 63.47 4.35 13.05 (63.51) (4.60) (13.29) |
| 5 | 3198($NH_2$),3178,3085(NH), 1615(C=N), 515(M-N) | 9468,16739,22160 | 43.48 2.81 22.53 (43.62) (3.06) (22.46) |
| 6 | 3190($NH_2$),3168,3080(NH), 1610(C=N), 512(M-N) | 9522,16290,21795 | 46.07 2.98 17.90 (46.32) (3.22) (17.75) |
| 7 | 3198($NH_2$),3165,3085(NH), 1612(C=N), 520(M-N) | 9520,15811,21448 | 52.08 3.03 18.21 (51.93) (3.16) (18.28) |
| 8 | 3205($NH_2$),3177,3085(NH), 1610(C=N), 515(M-N) | 9477,15918,22150 | 53.79 4.07 17.10 (53.71) (3.84) (17.34) |
| 9 | 3205($NH_2$),3178,3075(NH), 1615(C=N), 515(M-N) | 9510,15897,22200 | 55.10 3.36 17.12 (55.32) (3.38) (17.39) |
| 10 | 3202($NH_2$),3180,3082(NH), 1615(C=N), 498(M-N) | 9550,16290,22166 | 57.56 3.51 13.42 (57.71) (3.62) (13.48) |
| 11 | 3190($NH_2$),3165,3080(NH), 1610(C=N), 520(M-N) | 9538,16611,21382 | 62.20 3.56 13.59 (62.47) (3.42) (13.66) |
| 12 | 3205($NH_2$),3165,3075(NH), 1610(C=N), 520(M-N) | 9350,15648,21570 | 63.02 4.32 12.96 (63.38) (4.17) (13.03) |
| 13 | 3195($NH_2$),3160,3090(NH), 1610(C=N), 520(M-N) | 9411,15895,22252 | 43.08 2.79 22.32 (43.41) (2.67) (22.28) |
| 14 | 3190($NH_2$),3168,3090(NH), 1610(C=N), 495(M-N) | 9375,15912,21381 | 45.62 2.95 17.72 (45.55) (3.18) (17.95) |
| 15 | 3203($NH_2$),3180,3082(NH), 1615(C=N), 529(M-N) | 9545,16285,22168 | 51.57 3.0 18.03 (51.76) (2.83) (18.12) |
| 16 | 3202($NH_2$),3175,3085(NH), 1612(C=N), 520(M-N) | 9319,16730,21880 | 53.29 4.03 16.94 (53.51) (4.36) (16.88) |
| 17 | 3198($NH_2$),3168,3085(NH), 1610(C=N), 495(M-N) | 9465,16198,21995 | 55.51 3.38 17.25 (55.80) (3.23) (17.46) |
| 18 | 3190($NH_2$),3175,3085(NH), 1615(C=N), 520(M-N) | 9280,16511,21795 | 58.01 3.54 13.52 (58.39) (3.58) (13.83) |
| 19 | 3198($NH_2$),3168,3080(NH), 1610(C=N), 520(M-N) | 9485,16848,22258 | 62.69 3.58 13.70 (62.71) (3.33) (13.93) |
| 20 | 3205($NH_2$),3165,3085(NH), 1610(C=N),495(M-N) | 9455,16733,21448 | 63.50 4.35 13.06 (63.64) (4.68) (13.12) |
| 21 | 3202($NH_2$),3177,3085(NH), 1615(C=N),520(M-N) | 9468,16215,22531 | 43.50 2.81 22.54 (43.22) (3.06) (22.47) |
| 22 | 3202($NH_2$),3177,3085(NH), 1615(C=N),498(M-N) | 9550,15645,22200 | 46.09 2.98 17.91 (46.23) (3.24) (17.86) |
| 23 | 3190($NH_2$),3178,3085(NH), 1615(C=N),520(M-N) | 9290,16385,21765 | 52.11 3.03 18.22 (52.37) (2.83) (18.35) |
| 24 | 3195($NH_2$),3180,3075(NH), 1610(C=N),520(M-N) | 9535,16660,21570 | 53.82 4.07 17.11 (53.91) (4.18) (17.36) |

Three bands observed at 9915-11504, 16148-16210 and 2508-25580 $cm^{-1}$ in the spectra of the nickel(II) chelates are due to three spin-allowed transitions assignable respectively to $^3A_{2g}(F) \rightarrow {}^3T_{2g}$ (F)($V_1$), $^3A_{2g}(F) \rightarrow {}^3A_{1g}$ (F)($V_2$) and $^3A_{2g}(F) \rightarrow {}^3T_{1g}$ (P)($V_3$) consistent with idealised octahedral configuration[38].

In view of the above observations, it is proposed that all the metal chelates show an octahedral geometry by accomodating two ligands acting as tridentates and form a stable configuration of the metal chelate.

### Antibacterial properties

Our previous studies[39-43] in enlightening the role of metal ions give a detailed and systematic description of their antibacterial properties. However, in the present work, elaboration of the participating role of anions in the antibacterial activity is worked out. Table 4 reproduces the result of these studies. It is interesting to note that when the metal chelate having the same metal atom but different anions (nitrate, sulphate, oxalate or acetate) was individually screened for its antibacterial activity, its degree of potency killing the tested bacterial strains varied.

**Table 4** Antibacterial Activity Data

| Ligands/ Chelates | M i c r a | o b i a l b | S p e c c | i e s d |
|---|---|---|---|---|
| $L_1$ | ++ | + | ++ | ++ |
| $L_2$ | ++ | ++ | - | + |
| 1 | ++++ | +++ | ++++ | ++++ |
| 2 | +++ | ++ | + | +++ |
| 3 | ++++ | +++ | +++ | +++ |
| 4 | +++ | +++ | ++ | +++ |
| 5 | ++++ | +++ | +++ | ++++ |
| 6 | ++ | +++ | + | ++ |
| 7 | +++ | ++++ | +++ | +++ |
| 8 | +++ | +++ | ++ | +++ |
| 9 | ++++ | +++ | +++ | ++++ |
| 10 | ++ | +++ | + | ++ |
| 11 | +++ | ++++ | ++ | ++++ |
| 12 | +++ | +++ | ++ | +++ |
| 13 | ++++ | +++ | ++++ | +++ |
| 14 | +++ | ++ | + | ++ |
| 15 | ++++ | +++ | +++ | ++++ |
| 16 | +++ | ++ | ++ | +++ |
| 17 | ++++ | ++++ | ++++ | +++ |
| 18 | ++ | +++ | ++ | +++ |
| 19 | +++ | +++ | ++++ | +++ |
| 20 | +++ | ++ | +++ | +++ |
| 21 | ++++ | ++++ | +++ | ++++ |
| 22 | ++ | ++ | ++ | +++ |
| 23 | +++ | ++++ | ++ | ++++ |
| 24 | ++++ | +++ | + | ++++ |

a = *Staphylococcus aureus*, b = *Pseudomonas aeruginosa*, c = *Kliebsiella pneumonae*, d = *Proteus vulgarus*; Inhibition zone diameter (mm) +, 6-10; ++, 10-14; +++, 14-18; ++++, 18-22.

For example, cobalt chelate having nitrate anion was found to be more bactericidal than the cobalt chelate having anions sulphate, oxalate and acetate. The identical results were found for the other copper(II) and nickel(II) chelates. On comparison of the present data with the data obtained earlier for the same metal chelates of chloride anion, we are now, able to draw the order of potency as to be : nitrate > oxalate > acetate > chloride > sulphate which significantly alters the role of metal ions in the biological activity. We are, however, not able to explain at this stage, the possible mechanism of this role of anions. But our *in vitro* studies are in progress which may help us in determining this mechanism and the exact participating behaviour of anions.

### REFERENCES

1. J. R. Dilworth, *Coord. Chem Rev.*, **21**. 29(1976).
2. M. Katyal and Y. Dutta, *Talanta.*, **22**. 151(1975).
3. J. R. Merchant and D. S. Chothia, *J. Med. Chem.*, **13**, 335(1970).
4. K. Redda, L. A. Corleto and E. E. Knaus, *J. Med. Chem.*, **22**, 1079(1979).
5. M. F. Iskandar, S. E. Zayan, M. A. Khalifa and L. Ei-Sayed, *J. Inorg. Nucl. Chem.*, **36**, 556(1974).
6. R. C. Aggarwal, N. K. Singh and R. P. Singh, *Inorg. Chem.*, **20**, 2794(1981).
7. B. Singh, R. N. Singh and R. C. Aggarwal, *Synth. React. Inorg. Met-Org. Chem.*, **14**, 815(1984).
8. M. F. Iskandar, L. Ei-Sayed, A. F. M. Hefny and S. E. Zayan, *J. Inorg. Nucl. Chem.*, **38**, 2209(1976).
9. Z. H. Chohan and S. K. A. Sherazi, *Metal-Based Drugs.*, **4**, 69(1997).
10. Z. H. Chohan and A. Rauf, *Synth. React. Inorg. Met-Org. Chem.*, **26**, 591(1996).
11. Z. H. Chohan and S. K. A. Sherazi, *Metal-Based Drugs.*, **4**, 65(1997).
12. M. Gielen, *Coord. Chem. Rev.*, **151**, 41(1996).
13. M. Gielen, E. R. T. Tiekink, A. Bouhdid, D.de vos, M. Biesemans, I. Verbruggen and R. Willem, *Appl. Organomet. Chem.*, **9**, 639(1995).
14. E. B. Boyar and S. D. Robinson, *Coord. Chem. Rev.*, **50**. 109(1983)..
15. L. D. Dale, T. M. Dyson, D. A. Tocher and D. I. Edwards, *Anticancer Drug Res.*, **22**, 397(1989).
16. I. H. Hall, K. G. Rajendran, D. X. West and A. E. liberta, *Anticancer Drugs.*, **4**, 231(1993).
17. R. J. P. Williams, *Quart. Rev. Chem. Soc.*, **24**. 331(1970).
18. D. R. Williams, "The Metals of Life", Van Nostrand, London, 1971.
19. H. Sigel and D. B. McCrnic, *Accounts. Chem Res.*, **3**, 201(1970).
20. M. J. Seven and L. A. Johnson, "Metal Binding in Medicine", Lippincott Co, Philladelphia, PA, 1960.
21. Z. H. Chohan and M. A. Farooq, *J. Chem.Soc. Pak.*, **17**, 14(1995).
22. Z. H. Chohan and M. A. Farooq, *Pak. J. Pharmaceut. Sci.*, **7**, 45(1994).
23. Z. H. Chohan and H. Pervez, *Synth. React. Inorg. Met-Org. Chem.*, **23**, 1061(1993).
24. Z. H. Chohan and S. Kausar, *Chem Pharm. Bull.*, **41**. 951(1993).
25. Z. H. Chohan and S. Kausar, *Chem. Pharm. Bull.*, **40**. 2555(1992).
26. Z. H. Chohan and A. Rauf, *J. Inorg. Biochem.*, **46**, 41(1992).
27. D.H. William and I. Fleming, "Spectroscopic Methods in Organic Chemistry", 4th Ed, McGraw Hill Company, London, 1989.
28 M. Shallary, M. M. Moustafa and M. M. Bekheit, *J. Inorg. Nucl. Chem.*, **41**, 267(1979).
29. W. J. Geary, *Coord. Chem. Rev.*, **7**, 81(1971).
30. M. D. Glick and R. L. Lintvedt, *Prog. Inorg. Chem.*, **21**, 233(1976).
31. B. N. Figgis and J. Lewis, Prog. *Inorg. Chem.*, **6**, 37(1964).
32. L. J. Bellamy, "The Infrared Spectra of Complex Molecules", J. Wiley, N. York, 1971.
33. K. Nakamoto, "Infrared and Raman Spectra of Inorganic and Coordination Compounds", J. Wiley, N. York, 1978.
34. A. D. Liehr, *J. Phys. Chem.*, **67**, 1314(1967).
35. R. L. Carlin, "Transition Metal Chemistry", Ed, R. L. Carlin, Marcel Decker, N. York, Vol 1, 1965.
36. A. B. P. Lever, "Inorganic Electronic Spectroscopy", Elsevier, Amsterdam, 1968.
37. D. W. Meak, R. S. Drago and T. S. Piper, *Inorg. Chem.*, **1**, 285(1962).
38. B. N. Figgis and J. Lewis, "Modern Co-ordination Chemistry", Interscience, N. York, Ed, T. Lewis and B. R. G. Wilkin, 1960.
39. Z. H. Chohan and H. Pervez, *J. Pharmaceut. Sci.*, **6**, 17(1993).
40. Z. H. Chohan and S. Siddiqui, *Pak. J. Sci. Ind. Res.*, **36**, 132(1993).
41. Z. H. Chohan and H. Pervez, *J. Natur. Sci. Math.*, **22**, 241(1992).
42. Z. H. Chohan, *Chem. Pharm. Bull.*, **39**, 1578(1991).
43. Z. H. Chohan and A. Khaliq, *Pak. J. Med. Res.*, **28**, 92(1989).

**Received: October 14, 1997 - Accepted: December 3, 1997 - Received in revised camera-ready format: December 4, 1997**

